# Predictable regulation of survival by intratumoral microbe-immune crosstalk in patients with lung adenocarcinoma

**DOI:** 10.15698/mic2024.02.813

**Published:** 2024-02-19

**Authors:** Shuo Shi, Yuwen Chu, Haiyan Liu, Lan Yu, Dejun Sun, Jialiang Yang, Geng Tian, Lei Ji, Cong Zhang, Xinxin Lu

**Affiliations:** 1 The First Affiliated Hospital of Guangxi Medical University, Nanning 530021, Guangxi, China.; 2 Geneis Beijing Co., Ltd., Beijing 100102, China.; 3 Qingdao Geneis Institute of Big Data Mining and Precision Medicine, Qingdao 266000, Shandong, China.; 4 College of Information Engineering, Changsha Medical University, Changsha 410219, Hunan, China.; 5 Academician Workstation, Changsha Medical University, Changsha 410219, Hunan, China.; 6 Clinical Medical Research Center, Inner Mongolian People's Hospital, No. 20, Zhaowuda Road, Hohhot, Inner Mongolia, China.; 7 Inner Mongolia Key Laboratory of Gene Regulation of The Metabolic Disease, Inner Mongolian People's Hospital, No. 20, Zhaowuda Road, Hohhot, Inner Mongolia, China.; 8 Inner Mongolia Academy of Medical Sciences, Inner Mongolian People's Hospital, No. 20, Zhaowuda Road, Hohhot, Inner Mongolia, China.; 9 Pulmonary and Critical Care Medicine, Inner Mongolian People's Hospital, No. 20, Zhaowuda Road, Saihan District, Hohhot, Inner Mongolia, China.; 10 Hospital of Chengdu University of Traditional Chinese Medicine/No. 39, 12th Bridge Road, Jinniu District, Chengdu City, Sichuan Province, 610072, China.; 11 Nanjing Medical University Affiliated Cancer Hospital & Jiangsu Cancer Hospital & Jiangsu Institute of Cancer Research.

**Keywords:** lung adenocarcinoma, intratumoral microbiota, tumor microenvironment, immune cell, prognosis

## Abstract

Intratumoral microbiota can regulate the tumor immune microenvironment (TIME) and mediate tumor prognosis by promoting inflammatory response or inhibiting anti-tumor effects. Recent studies have elucidated the potential role of local tumor microbiota in the development and progression of lung adenocarcinoma (LUAD). However, whether intratumoral microbes are involved in the TIME that mediates the prognosis of LUAD remains unknown. Here, we obtained the matched tumor microbiome and host transcriptome and survival data of 478 patients with LUAD in The Cancer Genome Atlas (TCGA). Machine learning models based on immune cell marker genes can predict 1- to 5-year survival with relative accuracy. Patients were stratified into high- and low-survival-risk groups based on immune cell marker genes, with significant differences in intratumoral microbial communities. Specifically, patients in the high-risk group had significantly higher alpha diversity (p < 0.05) and were characterized by an enrichment of lung cancer-related genera such as *Streptococcus*. However, network analysis highlighted a more active pattern of dominant bacteria and immune cell crosstalk in TIME in the low-risk group compared to the high-risk group. Our study demonstrated that intratumoral microbiota-immune crosstalk was strongly associated with prognosis in LUAD patients, which would provide new targets for the development of precise therapeutic strategies.

## INTRODUCTION

Lung cancer (LC) is one of the most common malignancies and a leading cause of disease-related death around the world [1]. Histopathological differences divide LC into non-small cell lung cancer (NSCLC) and small cell lung cancer (SCLC). Lung adenocarcinoma (LUAD) is the most prominent cytological type of NSCLC, accounting for approximately 40% of LC cases [2]. Although multimodal treatment strategies including immunotherapy, targeted therapy, chemoradiotherapy, and surgical resection have made great progress in recent decades [3, 4], the 5-year survival rate for patients with LC remains below 20% [5, 6]. Therefore, it is urgent to clarify the pathogenesis, diagnostic biomarkers, and therapeutic targets of LC to facilitate the diagnosis and treatment of LC.

The tumor immune microenvironment (TIME) largely determines the prognosis and the effect of immunotherapy of patients with cancer [7–10]. The composition of the tumor microenvironment varies by tumor type, but signature features include immune cells, stromal cells, blood vessels, and extracellular matrix, and are generally recognized as active agents of cancer progression [11]. Tumors are infiltrated by a variety of adaptive and innate immune cells that can perform both pro- and anti-tumor functions [12]. Song *et al.* developed a seven-gene prognostic signature based on nature killer cell marker genes in The Cancer Genome Atlas (TCGA) LUAD cohort, and its ability to predict prognosis has been well validated in different cohorts [13]. One study quantitatively analyzed the immune cell infiltration across 32 cancer types and observed considerable heterogeneity in the prognostic correlation of these cells across different cancer types, and in particular, established an immune-cell characteristic score model for LUAD that had a favorable prognostic performance [14]. Although the effects of immune infiltration on cancer treatment and prognosis have been extensively studied [7, 9, 10, 15], the factors that influence immune infiltration and the contributing factors to the individual heterogeneity of TIME have been largely unknown.

The TIME provides a friendly niche for the presence of a wide range of microbes, and tissue-specific intracellular microbes have been identified in most human tumors [16]. The lungs of healthy individuals have long been considered sterile, but with the maturity of second-generation sequencing technology, the diversity of the lung microbiota and its relationship to lung disease and LC has been confirmed [17, 18]. Over the past decade, microbial communities have been implicated in the initiation, progression, metastasis, and response to treatment of a variety of cancers [19–22]. Recent studies have shown that microbes exist in tumor cells and immune cells, indicating that these microbes can affect the status of tumor immune microenvironment [23–25]. Studies have shown differences in the lung microbiome between patients with LC and those with benign lung disease, and that certain bacteria may have the potential to predict LC [26]. During the development of lung cancer, the number and species of commensal microorganisms in the lung changed, which promoted the proliferation and function of resident immune cells in the lung, furthermore, it promotes the development of LC through its effect on inflammatory reaction [27]. However, whether the microbiome in tumor tissue is related to the TIME and prognosis of LUAD remains unclear. Besides, the pattern of microbe-immune cell crosstalk in the TIME of LUAD and its prognostic implications need exploration. The whole-transcriptome sequencing data provided by TCGA offers a good opportunity to explore the crosstalk between the intratumoral microbiota and the TIME, which can be quantified based on host gene expression. Here, we identified immune cell marker genes in LC tissues and correlated them with the prognosis of patients with LUAD, and compared the intratumoral microbiota of high- and low-risk patients, as well as crosstalk patterns with immune cells in the TIME. We found that the immune cell marker gene-based machine learning model can predict the survival of patients with LUAD accurately. The intratumoral microbiota differed significantly between high- and low-risk patients, and there was variation in the crosstalk pattern between the microbial components and immune cells in the TIME.

## RESULTS

### Pipeline of this study

**Figure 1:**
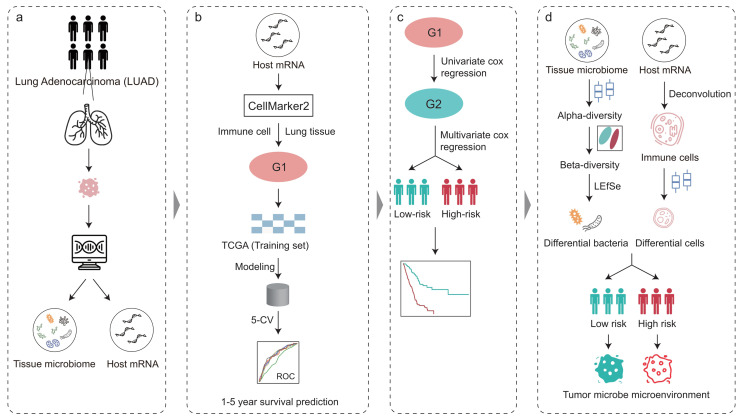
FIGURE 1: Overview of the analysis pipeline. The tumor microbiome abundance of LUAD was annotated by Poore *et al.* from RNA-Seq data and the matched host gene expression was downloaded from The Cancer Genome Atlas. Immune cell marker genes were used to build machine learning models to predict patient survival. COX regression analysis based on immune cell marker genes stratified patients into high- and low-risk. The intratumoral microbiota, the tumor immune microenvironment, and their crosstalk between the high-and low-risk groups were further explored.

The workflow of this study is shown in **Figure 1**. To illuminate the intratumoral microbiota in LUAD, we revisited and obtained the intratumoral microbial profiles in multiple cancer types, which were processed by Poore *et al.* using sequencing data in TCGA [28]. The LUAD samples in TCGA consist of 478 RNA sequencing (RNA-seq) data from the primary tumor of 478 patients. In addition, we also obtained the host gene expression of these patients with LUAD in TCGA which matched with the tumor microbiome.

**Figure 2:**
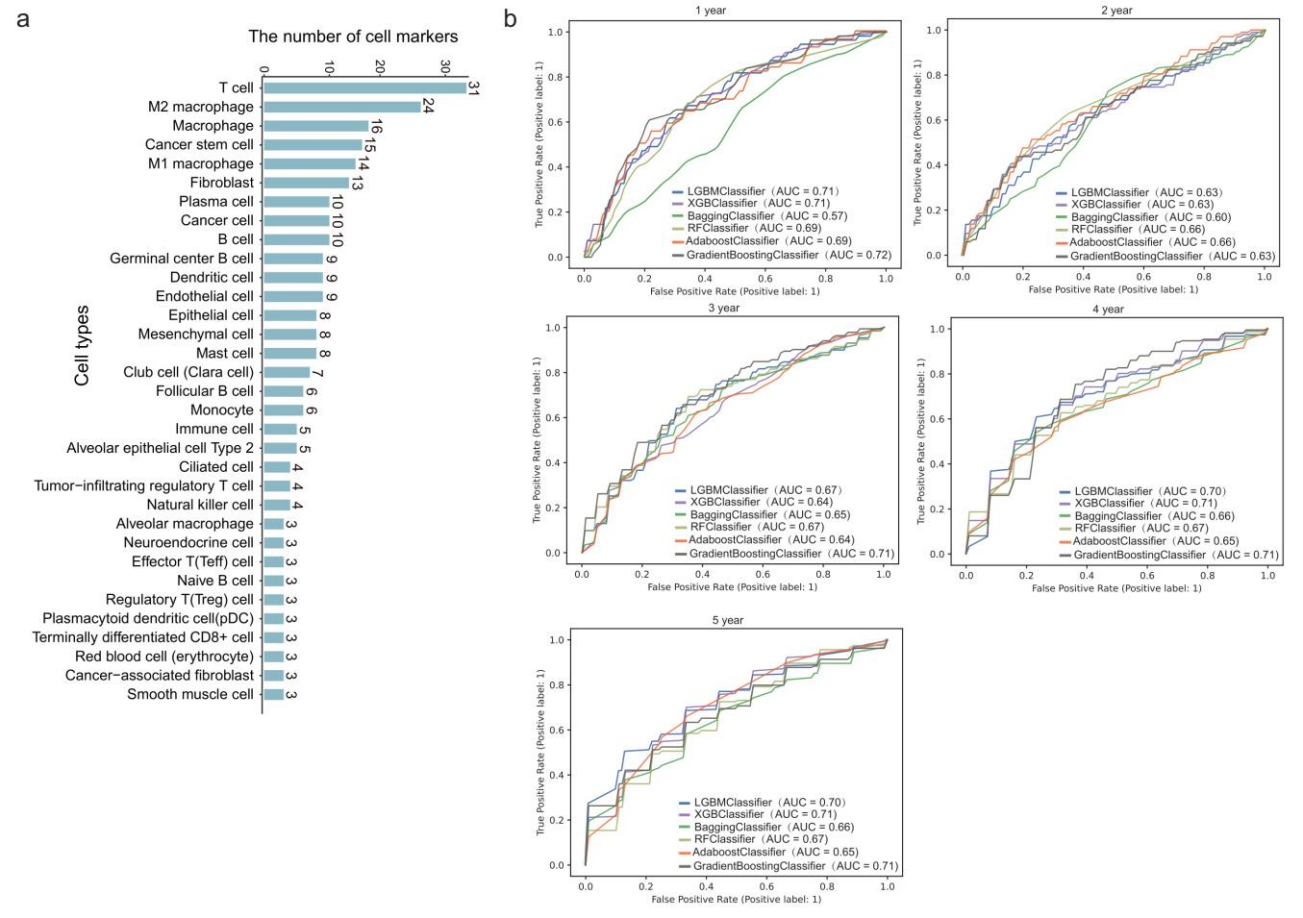
FIGURE 2: Survival prediction model based on immune cell marker genes using machine learning. **(A)** The number of marker genes corresponding to specific immune cell types. **(B)** ROC curves of 1- to 5-year survival prediction by five-fold cross validation of six machine learning algorithms.

We next downloaded cell marker genes from CellMarker2 database and selected lung tissue of LUAD to get marker genes in lung cells. By filtering out genes unrelated to immunity, we obtained 297 immune cell marker genes in lung tissue associated with LUAD. These genes corresponded mainly to 33 types of immune cells (**Figure 2A**). Among these, T cells had the most marker genes, reaching 31, followed by macrophages and cancer stem cells. Other cell types such as effector T cells, naive B cells, and alveolar macrophages (AM) etc. had only three marker genes. These genes will be used to predict the survival time of patients with LUAD.

### Immune cell marker genes show strong power in prediction of LUAD survival

478 patients with LUAD with their gene expression have been obtained from TCGA. First, we dichotomized patients based on survival time of one to five years, respectively. Five-fold cross validation was implemented to verify the accuracy of the six machine learning algorithms. Specifically, we classified all patients into five groups. Four of five group samples were used to train the model and the remaining one was used to test the model. After repeating this process five times, each group has been tested once and trained four times. As shown in **Figure 2B**, the prediction accuracy fluctuated slightly with the different survival time as the grouping threshold, and the difference of prediction accuracy of different algorithms was also very small. In some models, such as GB (gradient boosting), the mean AUC (area under the curve) for predicting one-year survival was up to 0.72, which means it is effective to predict the survival time of patients with LUAD through 297 immune cell marker genes.

### Immune-related activities are associated with survival in LUAD patients

**Figure 3:**
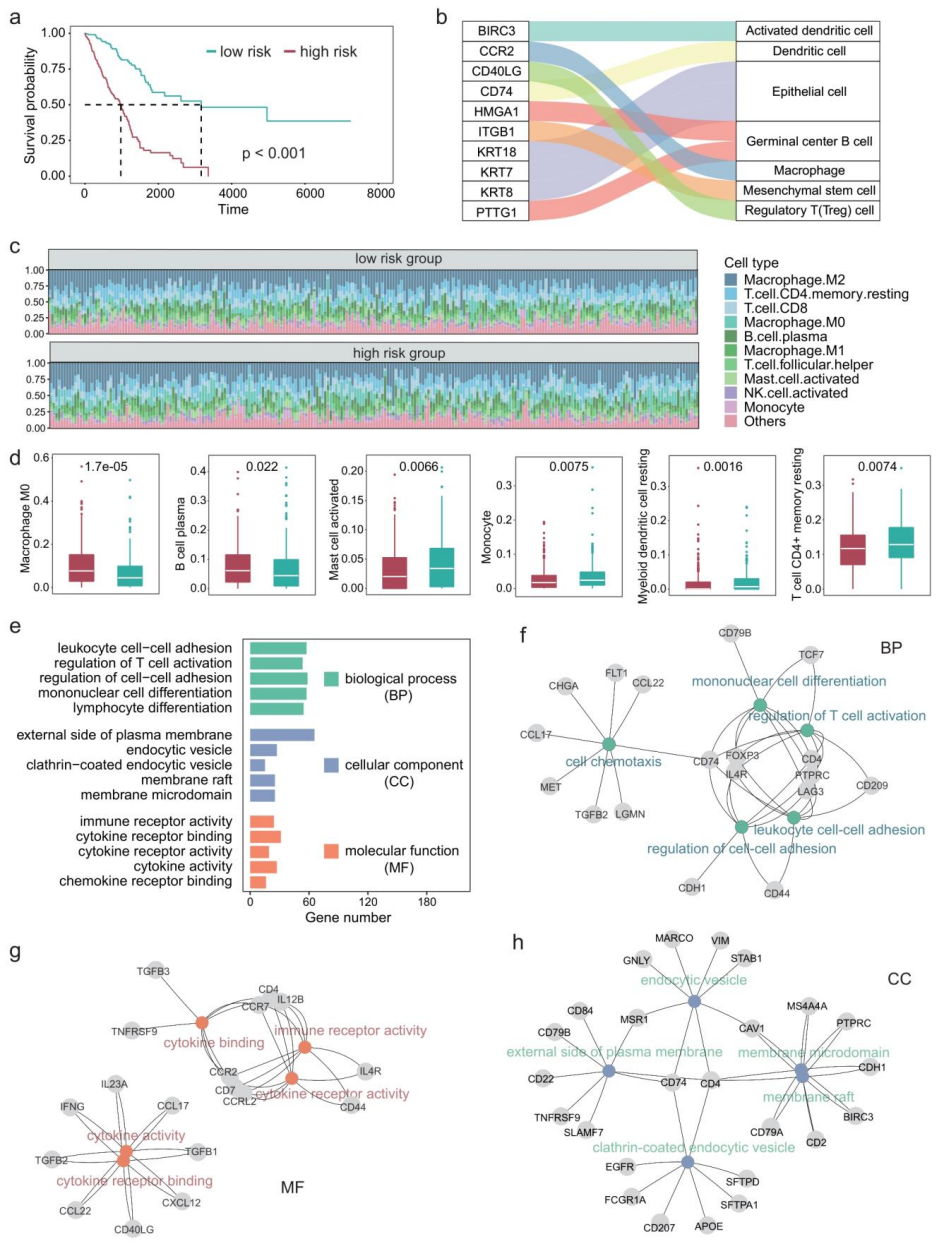
FIGURE 3: Tumor immune microenvironment and related functions are associated with survival in LUAD patients. **(A)** Survival curve in different risk score groups obtained by COX regression analysis. **(B)** Sankey plot showing the correlation between immune cells and the ten genes most associated with survival. **(C)** Relative abundance of the immune cell components in each patient. **(D)** Boxplot showing the differences in the abundance of immune cells between the high- and low-risk group. Wilcoxon test was used to perform the statistical test. **(E)** Five GO terms with the largest number of genes in each class. **(F-H)** The most significantly enriched GO terms in each class, along with the corresponding genes.

Since the TIME plays an important role in the development of LUAD [29, 30], we next explored the impact of differential expression of immune cell marker genes on the TIME of patients with LUAD. First, we performed univariate COX regression on 297 immune cell marker genes and identified 84 genes that were significantly associated with patient survival. Multivariate COX regression analyses were then performed based on these survival-related genes. The regression coefficients of these prognostic genes were obtained and the risk score of each patient was calculated based on the expression levels and coefficients of each gene. **Figure 3A** shows the survival curves of high-risk and low-risk. A P-value of less than 0.001 indicates that these prognosis-related immune cell marker genes could significantly distinguish the survival time of patients (**Figure 3A**). Furthermore, we examined the ten genes most significantly associated with survival and found that they corresponded to immune cell types such as macrophages and regulatory T cells (**Figure 3B**).

We next characterized the immune cell infiltration of patients in high-risk and low-risk. CIBERSORT was used to quantify the abundance of 22 types of immune cells in the TIME (**Figure 3C**). Among them, M2 macrophages had the highest abundance, followed by CD4+ T cells and CD8+ T cells. Moreover, we identified six types of immune cells that significantly differed between the high-risk group and the low-risk group, including M0 macrophages, B cell plasma, and myeloid dendritic cells (**Figure 3D**).

Since we used 297 genes to predict the survival time of patients with LUAD and the experiments results showed relatively high precision, these 297 genes should be significantly associated with the prognosis of LUAD in function. Therefore, we next explored the GO terms of these genes. Enrichment analysis showed that these genes were significantly associated with 1359 GO terms (adjusted P < 0.05). The GO terms can be divided into three classes: 1220 biological process, 59 cellular components, 80 molecular function. **Figure 3E** shows the five GO terms with the largest number of genes in each class. These genes enriched processes are associated with immune-related activities such as regulation of cell-cell adhesion, regulation of T cell activation, and cytokine receptor binding. **Figure 3F-H** showed the most significantly enriched GO terms in each class, along with the corresponding genes. CD74 is a receptor for the cytokine macrophage migration inhibitor [31], and Kashima *et al.* reported that CD74 is a novel gene that plays a key role in the drug-resistant state [32]. FOXP3 is a member of the forkhead transcription factor family, which is primarily expressed in a subset of CD4 + T cells and plays an inhibitory role in the immune system [33]. Yang *et al.* reported that FOXP3 can act as a co-activator of the Wnt-b-catenin signaling pathway, inducing epithelial-mesenchymal transition and tumor growth and metastasis in NSCLC [34]. Takanami found that CCR7 may be involved in the development of lymph node metastasis in NSCLC [35].

### Intratumoral microbiota differentiation between high- and low-survival-risk patients

**Figure 4:**
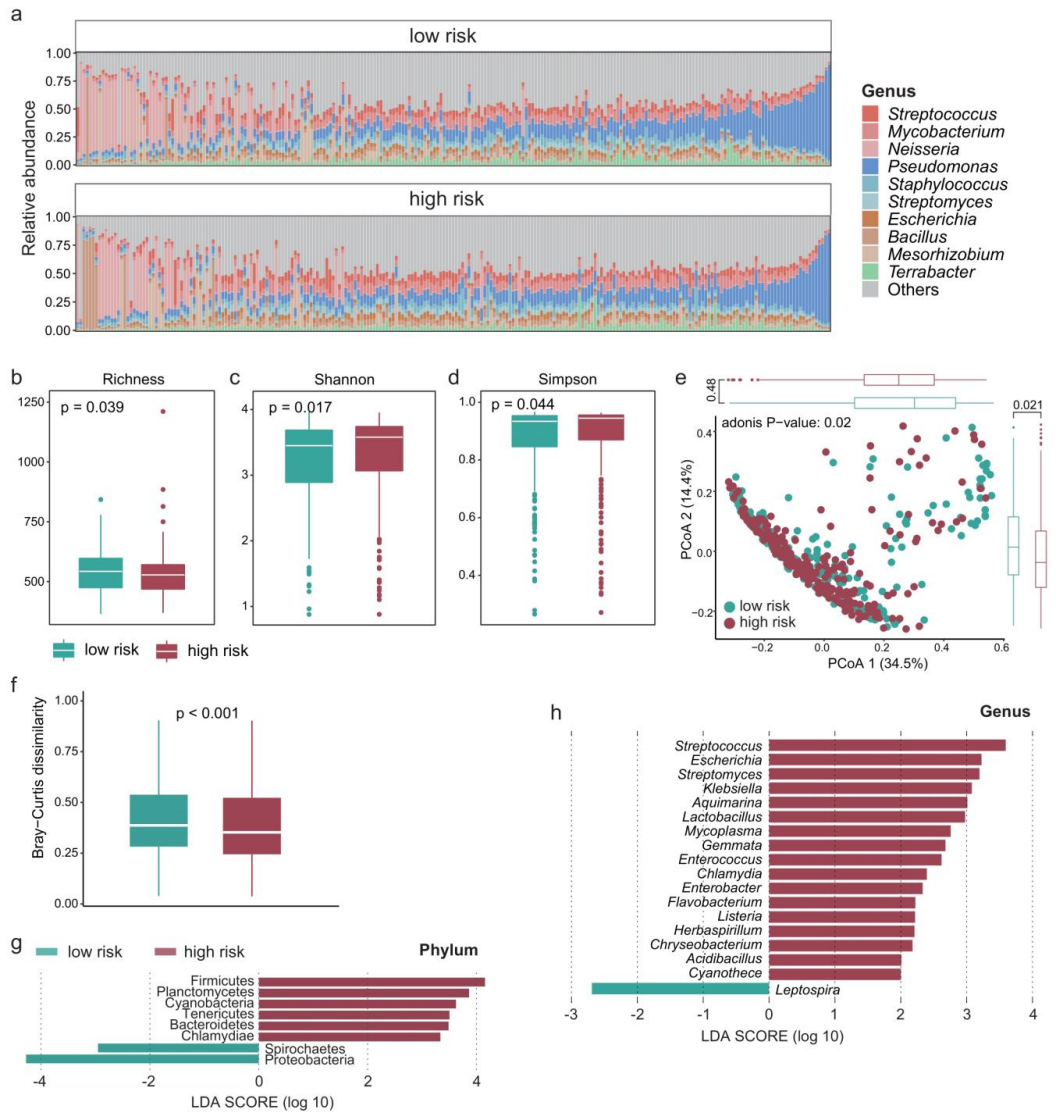
FIGURE 4: The intratumoral microbial profile was significantly different between the high- and low-risk group. **(A)** Relative abundance of the intratumoral microbes at the genus level in each patient. Boxplot showing the difference in **(B)** microbial richness, **(C)** the Shannon and **(D)** Simpson indices between the high- and low-risk group. Wilcoxon test was used to perform the statistical test. **(E)** PCoA based on the Bray-Curtis dissimilarity matrix showing the difference in intratumoral microbial community composition between the high- and low-risk group. **(F)** Boxplot showing the difference in Bray-Curtis dissimilarity index between the high- and low-risk group. Significantly different microbes in abundance between the high- and low-risk group at the **(G)** phylum and **(H)** genus level.

Recent studies have shown that the intratumoral microbiota plays a key role in theTIME [23, 36], so we next explored whether there are differences in the intratumoral microbiota between high- and low-survival-risk patients. *Proteobacteria* was the most abundant phylum and *Pseudomonas* was the most abundant genus (**Figure 4A**). There was significant difference in alpha-diversity between high- and low-survival-risk patients (P < 0.05). For instance, the microbial abundance of the low-risk group was significantly higher that of the high-risk group (**Figure 4B**, P = 0.039), while the Shannon (**Figure 4C**, P = 0.017) and Simpson indices (**Figure 4D**, P = 0.044) of the low-risk group were significantly lower than that of the high-risk group. Moreover, beta-diversity analysis showed that intratumoral microbial profiles were significantly different between the low-risk and high-risk group (**Figure 4E**, P = 0.02), and beta-diversity was more dissimilar among individuals in the low-risk group (**Figure 4F**, P < 0.001). Based on linear discriminant analysis effect size (LEfSe) analysis, at the phylum level, six phyla were enriched in the high-risk group and two phyla were enriched in the low-risk group (**Figure 4G**). At the genus level, 17 genera were enriched in the high-risk group and one genus was enriched in the low-risk group (**Figure 4H**).

### Different microbiota-immune crosstalk patterns in the TIME between the high- and low-risk group

**Figure 5:**
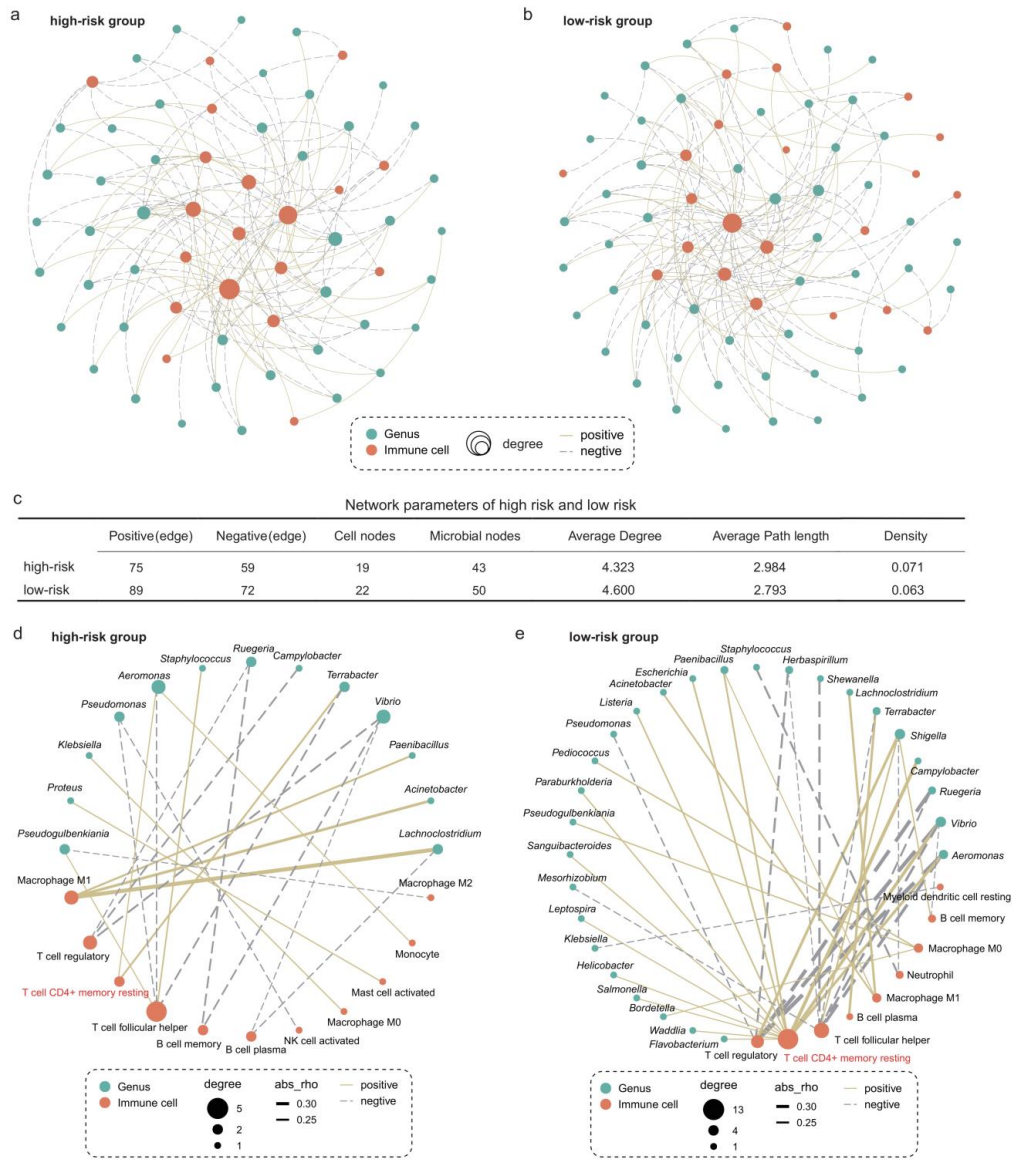
FIGURE 5: Intratumoral microbe-immune crosstalk was associated with survival in LUAD patients. Microbe-immune cell interaction networks in (A) high- and (B) low-risk groups. Only edges with p < 0.05 were shown in the figure. The size of the node indicates the number of nodes connected to it in the network. The solid yellow line and the dotted gray line indicate positive and negative correlations, respectively. (C) Comparison of parameters of microbe-immune interaction network in high- and low-risk group. On the basis of Figure 5a and b, microbial-immune cell relationship pairs with an absolute value of correlation coefficient greater than 0.2 were screened, further resulting in network plots of (D) high- and (E) low-risk groups.

We next explored whether microbiota-immune cell crosstalk in the TIME differed between the high- and low-risk group. Considering the predominance of dominant bacteria in the community, we performed Spearman correlation analysis for the top 50 genera in relative abundance and 22 types of immune cells. **Figures 5A** and **B** show only the microbe-immune cell pairs that were significantly associated (p < 0.05). The results of network analysis showed that the low-risk group presented more active microbe-immune crosstalk than the high-risk group (**Figure 5A-C**). The high-risk network had 134 edges, including 75 positive correlations and 59 negative correlations, while the low-risk network had 161 edges, including 89 positive correlations and 72 negative correlations (**Figure 5C**). In addition, more nodes and higher average degree of nodes in the low-risk group network than in the high-risk group indicated more complex and robust microbe-immune crosstalk pattern in the low-risk group.

To further explore the relationship between specific microbes and specific cells, we screened the significantly correlated microbial-immune pairs with absolute correlation coefficients greater than 0.2 (**Figure 5D-E**). We observed some common and significant microbe-immune cell associations in the high-risk and low-risk groups. For instance, in both groups, *Lachnoclostridium*, *Acinetobacter*, and *Paenibacillus* were positively correlated with M1 macrophages. *Aeromonas* and *Vibrio* were negatively correlated with regulatory T cells. However, we still identified multiple correlations between microbes and cell types which were specific for the survival risk group. Most notably, memory CD4+ T cells were positively associated with more than a dozen bacteria in the low-risk group, compared with just two in the high-risk group. The role of memory T cells in LC has been extensively studied [37, 38]. One study reported that when tissue-resident memory T cells are present in tumors, they act together to attack the cancer cells and protect the host [38]. Our results show that the interaction between memory T cells and intratumoral bacteria in TIME is different in patients with LUAD with different survival risks.

## DISCUSSION

Recent studies have identified the presence of intratumoral microbiota in various non-gastrointestinal tumors, including LUAD. However, the role of the intratumoral microbiota in the prognosis of LUAD remains largely unknown. In this study, survival of patients with LUAD could be distinguished based on immune cell marker genes. The intratumoral microbiota varied between high- and low-survival-risk patients using these immune cell marker genes. Moreover, the intratumoral microbiota-immune cell crosstalk pattern were found to be different between these two groups, which may contribute to the prognosis of patients with LUAD.

In the experiment of predicting the survival of patients with LUAD by selected 297 immune cell marker genes, the machine learning model obtained the result of AUC = 0.72, which is relatively accurate, but there is still room for improvement. These immune cell-specific genes correspond to a wide variety of immune cells, not all of which are involved in the development of LUAD. Therefore, more mechanistic studies are needed to investigate and identify specific immune cells that may influence the progress of LUAD, or to identify tumor immune infiltration characteristics in patients with different prognostic risks. Therefore, predictive models based on marker genes of specific immune cells that regulate the development of LUAD through a well-defined mechanism or function can greatly improve the accuracy of patient prognosis prediction. Altogether, only by accurately identifying and classifying immune cell marker genes related to pathogenesis and treatment can achieve personalized and precise treatment.

The diversity of TIME profiles in patients with LUAD has been highlighted in previous reports, proving that it could serve as a hallmark for LUAD development [39–42]. Shinohara *et al.* conducted a single-sample gene set enrichment analysis of TIME-related gene sets to develop a new scoring system (TIME score), the TIME score captures the intricate interactions between tumor proliferation, anti-tumor immunity and immunosuppression, which may be useful in predicting the prognosis or selecting treatment strategies in patients with LC [42]. Taniguchi *et al.* revealed that AMs promote the proliferation of cancer cells [43]. Under tumor-containing conditions, the expression of statin βA (INHBA) in lung AMs is up-regulated, thus promoting tumor proliferation and forming a “vicious cycle” in *in vivo* tumor environment. We found that the abundance of M0 macrophages was significantly higher in the high-risk group compared to the low-risk group (p < 0.0001), consistent with previous reports. In addition, B and plasma cells (PCs) were found to be more abundant in the high-risk group. By a comprehensive analysis of 50,000 tumor-infiltrating B and PCs, Hao *et al.* found that memory B cells and PCs were highly enriched and highly differentiated in tumor tissues, and PC were significantly increased in smokers with distinct differentiation trajectories [44]. Furthermore, one study showed that memory T cells in lung tumors predicted good outcomes for patients, and that patients with high levels of these cells in their tumors were 34% less likely to die [38]. Consistently, we found that tissue-resident memory CD4+ T cells were more enriched in the low-risk group compared with the high-risk group.

We found that the intratumoral microbiome profiles were significantly different between the high- and low-risk group. Interestingly, a large number of genera were significantly enriched in the high-risk group, including multiple LC-related pathogens, such as *Streptococcus*, *Escherichia*, and *Klebsiella*. Li *et al.* reported that LC cells infected with *Streptococcus pneumoniae* formed larger tumors in mice compared to untreated LC cells, and their abundance was associated with survival [45]. LC surgery is prone to serious infectious complications caused by Gram-negative bacteria such as *Escherichia coli*, which may reduce long-term survival after discharge through cancer recurrence and metastasis [46]. *Klebsiella* expression is more pronounced in lung squamous-cell carcinoma than in LUAD, however, we still found significantly enriched *Klebsiella* in high-risk LUAD patients, suggesting their potential significance in LUAD prognosis [47]. Microbiota-immune crosstalk in the TIME may contribute to the heterogeneity of outcomes in patients with LUAD. Jin *et al.* reported that LC alters the number and type of microbes in the lung and activates the immune system, creating an inflammatory environment for LC and ultimately promoting the development of LC [27]. Although intratumoral microbial alpha diversity was significantly lower in low-risk patients than in high-risk patients, we identified more complex and close microbe-immune interactions in low-risk patients. Predictive models that combine immune cell marker genes with their associated intratumoral microbiota may further improve performance in predicting patient survival. In addition, our results suggest that targeting specific microbes within tumors can modify tumor immune infiltration by exploiting the association of microbes with immune components. Future multicenter studies with larger cohorts will be needed to determine the TIME characteristics that are most favorable to patient outcomes for LUAD.

There were several limitations in this study. A major limitation was that our study on the interactions between intratumoral microbiota and immune cells were only based on a single TCGA dataset, and lacked external independent verification. Moreover, the causal relationship and specific mechanisms between intratumoral microbiota and immune and LUAD prognosis require rigorous experimental verification. Another limitation was that the tumor microbiome abundance was obtained by Kraken pipeline from RNA sequencing data. Therefore, it is necessary to validate our results by other microbial detection methods, such as metagenomic sequencing or PCR analysis.

In conclusion, this study advances the understanding of the relationship between intratumoral microbe-immune crosstalk and prognosis in patients with LUAD. Although components in the TIME have emerged as potential targets for lung cancer immunotherapy, our study suggests that ignoring the important role of intratumoral microbiota in the TIME may not enable all patients to benefit from immunotherapy. Future development of emerging immunotherapy strategies for LUAD will require perturbation of the microbe-immune cell crosstalk pattern in the TIME to achieve truly individualized precision-targeted therapies.

## MATERIALS AND METHODS

### Data acquisition

The intratumoral microbiome data used in this study and the metadata were downloaded from a previous work conducted by Poore *et al.*, and are available at ftp://ftp.microbio.me/pub/cancer_microbiome_analysis/. Poore *et al.* developed a Kraken TCGA microbial-detection pipeline, which uses an ultrafast Kraken algorithm to map sequence readings that do not align with the human reference genome to known bacterial, viral, and archaea microbial genomes [28]. The bacterial abundance data in tumor tissue of patients with LUAD was used in this study. The decontamination process was detailed in the original paper [28]. The overall survival time and survival status of the samples were collected from UCSC Xena (http://xena.ucsc.edu/). The quantification of host gene expression by RNA-Seq were downloaded from https://portal.gdc.cancer.gov/.

### Selection of immune cell marker genes

The immune cell marker gene must satisfy two conditions: first, it must be the immune cell marker gene in the lung, and second, all the immune cell marker genes must be related to LUAD. By defining LUAD and immune cells, we obtained a total of 297 immune cell marker genes from the CellMarker 2.0 [48] database.

### Quantification of immune cells in the TIME of patients with LUAD

CIBERSORT [49] was used to perform the immune cell analysis based on the TCGA LUAD gene expression data. We converted FPKM (fragments per kilobase of transcript per million fragments mapped) value to TPM (transcripts per million) value because TPM can correct the batch effect, so the sum of FPKM is a fixed value.

### Survival time prediction method

Six machine learning models were used to predict the one to five-year survival of patients with LUAD. These models were implemented using the scikit-learn library in Python. Bagging (Bootstrap Aggregating) is an ensemble learning method that reduces model variance and improves prediction stability and accuracy by combining multiple decision trees. The fundamental idea is to randomly select multiple sub-samples from the training dataset using bootstrap sampling and train multiple base learners on these sub-samples. Finally, the predictions of these base learners were aggregated to obtain the final ensemble prediction.

LGBM, XGBoost (XGB), and GB (Gradient Boosting) are three machine learning algorithms based on Gradient Boosting Trees. The formula for the Gradient Boosting Tree algorithm is as follows:


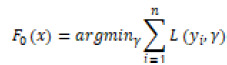



L(*y*_i_, γ) is the loss function, *y*_i_ is the true label of the first sample of the training data, and γ is the initial predicted value of the model. For the m-round iteration, a new decision tree model *h*_m_(x) is constructed on the basis of the previous round model, with the goal of reducing the loss function *r*_im_:


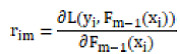



The newly constructed model *h*_m_(x) is weighted with the previous one *F*_m−1_(x) to get the updated model *F*_m−1_(x):


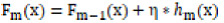



Among *η* is the learning rate, which can control the weight of each model. Iterative updates are repeated until a predetermined number of iterations is reached.

LGBM, XGBoost, and GB are three gradient boosting tree algorithms that optimize and enhance the gradient boosting algorithm, thereby improving model efficiency and prediction performance. On the other hand, Adaboost is a specialized implementation of gradient boosting trees. It iteratively trains a series of weak classifiers (usually decision trees) and calculates weights for each weak classifier based on its error rate, resulting in a strong classifier.

The advantages of Adaboost lie in its ability to effectively enhance classifier accuracy and handle complex problems. As an ensemble learning method, Random Forest predicts patient survival by constructing multiple decision trees, each trained on random samples of data and features. The predictions of multiple trees are then combined to obtain the final survival prediction. To evaluate the model performance and prevent overfitting, we employed 5-fold cross-validation. The average concordance index obtained from the 5-fold cross-validation served as the evaluation metric for the models.

### Microbial diversity analysis

The microbial alpha diversity was measured by the Shannon and Simpson indices, and was calculated by the “vegdist” function in R package “vegan”. The microbial beta diversity was measured by the Bray-Curtis dissimilarity matrix. Linear discriminant analysis Effect Size (LEfSe) was used to identify the significantly different microbes in relative abundance, with a linear discriminant analysis (LDA) score greater than 2 as the threshold.

### Construction of microbe-immune cell crosstalk network

To investigate the interactions between intratumoral microbes and immune cells, we conducted a correlation analysis on 22 types of immune cells and the top 50 microbes in relative abundance at the genus level. The “psych” package in R was used to perform the Spearman correlation analysis and calculate the correlation coefficients and p-values. First, to explore the overall properties of microbe-immune interaction networks in high-risk and low-risk groups, significant relationships with p-values < 0.05 were selected to construct the microbe-immune interaction network. Gephi was used to visualize the network. Furthermore, to identify the interaction of a particular microbe with a particular cell, pairs with an absolute value of correlation coefficient greater than 0.2 and a p value less than 0.05 were screened for further network construction.

### Statistical analysis

All statistical calculations were conducted using R software (Version 4.2.1). Differences between two groups were compared using Wilcoxon rank sum test. Correlations between immune cells and intratumoral microbes were calculated using Spearman's correlation analysis. Survival curves were performed using the Kaplan–Meier (KM) method, and the significance was determined by the log-rank test. Univariate Cox regression analysis was used to calculate the significance of the association between immune cell marker genes and prognosis in patients with LUAD. The Python package “Sklearn” and library “matplotlib” was used to plot receiver operating characteristic (ROC) curves and obtain the area under the curve (AUC). *p* < 0.05 was considered statistically significant.

### Data availability

The intratumoral microbiome abundance data and metadata are available at ftp://ftp.microbio.me/pub/cancer_microbiome_analysis/. The host gene expression data is available at https://portal.gdc.cancer.gov/.

## Abbreviations:

AM: – alveolar macrophage,
LC: – lung cancer,
LUAD: – lung adenocarcinoma,
NSCLC: – non-small cell LC,
PC: – plasma cell,
TCGA: – The Cancer Genome Atlas,
TIME: – tumor immune microenvironment.
